# Marek’s disease virus-specific T cells proliferate, express antiviral cytokines but have impaired degranulation response

**DOI:** 10.3389/fimmu.2022.973762

**Published:** 2022-09-15

**Authors:** Nitish Boodhoo, Shahriar Behboudi

**Affiliations:** Avian Immunology, The Pirbright Institute, Woking, United Kingdom

**Keywords:** T cells, genetic resistance, genetic susceptibility, Marek’s disease virus, immunodominant epitopes

## Abstract

The major histocompatibility complex (MHC) haplotype is one of the major determinants of genetic resistance and susceptibility of chickens to Marek’s disease (MD) which is caused by an oncogenic herpesvirus; Marek’s disease virus (MDV). To determine differential functional abilities of T cells associated with resistance and susceptibility to MD, we identified immunodominant CD4+TCRvβ1 T cell epitopes within the pp38 antigen of MDV in B19 and B21 MHC haplotype chickens using an *ex vivo* ELISPOT assay for chicken IFN-gamma. These novel pp38 peptides were used to characterize differential functional abilities of T cells as associated with resistance and susceptibility to MD. The results demonstrated an upregulation of cytokines (IL-2, IL-4, IL-10) and lymphocyte lysis-related genes (perforin and granzyme B) in an antigen specific manner using RT-PCR. In the MD-resistant chickens (B21 MHC haplotype), antigen-specific and non-specific response was highly skewed towards Th2 response as defined by higher levels of IL-4 expression as well as lymphocyte lysis-related genes compared to that in the MD-susceptible chicken line (B19 MHC haplotype). Using CD107a degranulation assay, the results showed that MDV infection impairs cytotoxic function of T cells regardless of their genetic background. Taken together, the data demonstrate an association between type of T cell response to pp38 and resistance to the disease and will shed light on our understanding of immune response to this oncogenic herpesvirus and failure to induce sterile immunity.

## Introduction

Marek’s disease (MD) is a severe and deadly lymphoproliferative disease of chickens that is widespread in the world (1, 2). The disease is caused by Marek’s disease virus (MDV), a *Gallid herpesvirus 2 (GAHV-2)*, which causes significant economic loss in poultry industry (1, 3). MD is associated with metabolic dysregulation (4–7), immunosuppression (8–14), neurological disorder (15), and CD4+ lymphoma formation in chickens (1, 16). Because of MDV’s highly cell associated nature, it is believed that virus-specific T lymphocytes are critical in control of MD (17, 18). Vaccination inhibits MD but fails to control MDV replication and shedding (19). The failure of vaccine-induced immunity to provide sterile immunity may contribute to the emergence of more virulent MDV. Unlike in mammals, the level of resistance to several infectious pathogens is determined with chicken MHC (encoded by the BF-BL region of the B locus). Although, it should be noted that other yet unknown factors may also be involved in resistance to MD, as some chicken lines (*e.g.* line 6 and line 7) with identical MHC molecules have different levels of susceptibility to MD. Chicken lines with B19 MHC haplotype (line P2a) are highly susceptible to MD, while those chickens with B21 MHC haplotype (line N) are resistant to MD (20–22). Thus, understanding differential functions of T cell responses in the MD-resistant and susceptible chickens may shed light on correlate of protection and development of more efficacious vaccines which may inhibit virus replication and shedding (23–25).

We have recently identified two immunogenic regions of pp38 recognized by T cells from the MD-susceptible B19 MHC haplotype and one immunogenic pp38 region recognized by T cells from the MD-resistant B21 MHC haplotype chicken lines (26). In the present study, we identified two B19 and one B21 restricted 15 mer immunodominant T cell epitopes within the immunogenic regions of *pp38* which are recognized by IFN-gamma producing CD4+ TCRνβ_1_+ T cells. Previously, we had demonstrated differential IFN-gamma expression by virus-specific memory T cells between the MD-resistant and susceptible chickens. Here, functional differences in cytokine and lymphocyte lysis-related genes in virus-specific effector T cells are found between the MD-resistant B21 MHC haplotype and MD-susceptible B19 MHC haplotype chicken lines. Moreover, the results demonstrate that MDV infection impairs degranulation of T cells regardless of their genetic background and MHC haplotype. Thus, this report identifies association between resistance to MD and differential functional abilities of virus-specific effector T cells and provides valuable information on T cell immunity against MD in chickens.

## Materials and methods

### Virus preparation

Chicken embryonic fibroblast cells (CEFs) were generated from ten-day-old mixed-sex specific pathogen free (SPF) Valo eggs (Valo Biomedia GmbH). CEFs were cultured in M199 medium (Life Technologies, Paisley, UK), supplemented with 5% fetal bovine serum (FCS) (Sigma, Dorset, UK), 100 units/mL of penicillin and streptomycin (Life Technology), 0.25 μg/mL Fungizone (Sigma, Dorset, UK), and 10% TPB (tryptose phosphate broth), (Sigma, Dorset, UK).Virus stocks were 3rd passage of the virulent MDV (GaHV-2: RB1B) and vaccine strain of MDV (CVI988/Rispens), propagated as cell-associated stocks in CEF for 72 hours. The virus stocks were titrated on fresh CEFs and the plaques were visualized using anti-gB mAb (HB-3) for staining. Commercial CVI988/Rispens vaccine virus (Nobilis Rismavac) was obtained from Intervet.

### Animal experiments

Genetically defined mixed sex specific pathogen free (SPF) chicken line N (MD-resistant; B^21^ MHC haplotype) and line P2a (MD-susceptible; B^19^ MHC haplotype) were purchased from National Avian Research Facility (NARF) at University of Edinburgh. Day old chicks were grouped housed throughout the experiment in specific pathogen free filtered-air positive pressure rooms on floor pens with wood shaving. Group housed chickens had ad libitum access to water and commercial feed. Line N and line P2a chickens were either mock inoculated (non-infected CEF), challenged (RB1B; 1,000 pfu/chicken) or vaccinated (infected with vaccine strain: CVI988/RispensS; 1,000 pfu/chicken) at 1 day of age *via* intra-abdominal route and the vaccinated group (infected with CVI988/Rispens) were either boosted with the vaccine strain of MDV (CVI988/Rispens; 1,000 pfu/chicken) or challenged with the virulent strain of MDV (RB1B; 1,000 pfu/chicken) 2 weeks later *via* intra-abdominal route. Splenocytes were harvested in PBS with penicillin (10 U/ml), and streptomycin (10 μg/ml) on ice at different time post infection for further analysis.

### Synthetic peptide library

In total, 20 peptides spanning two parts of the *pp38* of RB1B strain of MDV (GenBank: ABR13155.1), covering amino acid sequences between 1-60 and 151-211, were synthesized by Mimotopes (United Kingdom). There is no difference in amino acid sequences of pp38 between RB1B (virulent) and CVI988-Rispens (vaccine strain) within these two sections. We have recently shown that these immunogenic *pp38* sections of MDV may contain immunodominant epitopes (27) restricted to B19 and B21 MHC haplotypes (26). Peptides,15 residues long and overlapped by 10 residues, were dissolved in DMSO and peptide pool 1 (1-60 aa) peptide pool 4 (151-211 aa) with 10 peptides in each per pool were prepared.

### Spleen mononuclear cell preparation

Mononuclear cells were isolated from chicken spleens as previously described (27). In brief, spleens were crushed onto 40-μm BD cell strainers (BD Biosciences, UK), and the collected cells were layered on LymphoprepTM (Axis-shield PoC AS, Norway) density-gradient, and centrifuged (500 x G for 30 min at 4°C). Mononuclear cells were isolated from the interface, washed (250 x Gfor 10 min at 4°C) and suspended in complete media containing Roswell Park Memorial Institute medium-1640 (RPMI-1640) supplemented with 10% foetal bovine serum (Sigma-Aldrich, Dorset, UK), penicillin (10 U/ml), and streptomycin (10 μg/ml). After determining cell viability by trypan blue exclusion method, the splenocytes count was adjusted to 5 x 10^6^ cells/ml.

### *Ex vivo* chicken IFN-γ ELISPOT assay

#### (i) αβ T cell depletion

Approximately 1.0x10^7^ splenocytes from each chicken were washed in buffer solution [Phosphate-buffered saline (PBS) + 0.5% fetal calf serum (FCS)] and incubated (4°C for 15 min) in the presence of anti-CD4-PE, anti- *CD8_β_*-PE, anti-TCRvβ_1_-PE or anti-TCRvβ_2_-PE antibody (Southern Biotech, UK). Mononuclear cells were washed (250 x G for 4 min) and incubated (4°C for 15 min) further with 85μl of buffer solution and 15 μl of anti-PE micro beads (Miltenyi Biotec, Surrey, UK). Microbead magnetic labelled mononuclear cells were applied to LS MACS column (Miltenyi Biotec, Surrey, UK) and the pass through collected. Depletion purity (>98%) was confirmed by flow cytometry using a MACSQuant 10 flow cytometer (Miltenyi Biotec). Mononuclear cells suspensions were washed again, re-suspended in complete RPMI 1640 medium at a rate of 3.3 × 10^6^ cells/ml and stored on ice until required.

#### (ii) *pp38*-specific effector T cell response

*pp38*-specific effector T cell response was evaluated based on the frequencies of IFN-γ producing T cells using an *ex vivo* chicken IFN-γ ELISPOT assay kit which includes all the antibodies (Life technologies, UK). In brief, MAIPS4510 MultiScreenTM-IP 96 well plates (Millipore, UK) were incubated overnight at 4°C with 2 μg/ml mouse anti-chIFN-γ (capture antibody) for 18 hrs. Mononuclear cells from each chicken were seeded in triplicates at a rate of 3.3 x 10^5^ cells per well and the cells were stimulated with *pp38* derived peptide pools (containing 1 μM of each individual peptide; 10 peptides in each peptide pool) or an irrelevant peptide (derived from influenza HA). In each assay, some cells were also incubated with diluent (negative control), Phorbol Myristate Acetate (PMA; 50 ng/ml) plus Ionomycin (Ion; 1 μg/ml) (positive control); (Sigma-Aldrich, Dorset, UK), at 41°C and 5% CO_2_ overnight. Next day, plates were washed twice with water and three times with washing buffer (PBS + 0.1% Tween 20). Plates were subsequently incubated with detection antibody (1 μg/ml of anti-chicken IFN-γ biotinylated antibody) and this was followed with Streptavidin- HRP (1/1250). The assay was developed in the presence of 3-Amino-9-ethylcarbazole (AEC) substrate solution (BD Biosciences, UK). The numbers of IFN-γ producing T cells (spots forming units; SFU) was determined using an automated ELISPOT reader. The SFU was calculated by subtracting the number of spots obtained in the non-stimulated control wells or irrelevant peptide pools from the stimulated samples.

### Flow cytometry

#### (i) Carboxy fluorescein diacetate, succinimidyl ester-based T cell proliferation assay

Approximately 1.0x10^7^ splenocytes from each chicken were labelled with CFSE (5 µM, eBioscience, UK) according to the manufacture’s recommendation. Staining was quenched in pre-warmed RMPI complete media (37°C for 30 min) and cell numbers adjusted to 5.0 x10^6^ cells/mL. CFSE-labelled splenocytes were stimulated in the presence pp38-derived peptides or an irrelevant peptide (influenza HA peptide: H5_246–260_). At 72 hrs post stimulation, splenocytes were washed [PBS + 0.5% Bovine serum albumin (BSA)] and counter stained with chicken anti-CD4-PE, anti-CD8_β_-APC (Cambridge Bioscience, Cambridge, UK) and dead cells marker (7AAD) (BD Bioscience, Oxford, UK). Cell proliferation and frequency of specific T cell subsets were detected by monitoring changes in fluorescence intensity of CFSE-labeled cells (FITC) at 72 hrs post stimulation.

#### (ii) CD107a degranulation assay

The supernatant of LEP100 hybridoma cells (Developmental Studies Hybridoma Bank, Iowa City, Iowa, USA) were collected and the anti-CD107a and isotype control monoclonal antibodies (mouse IgG1 isotype) were purified using the Protein G Chromatography Cartridge (Thermo Fisher Scientific, Paisley, UK) according to the manufacturer’s protocol. Column purified antibodies were conjugated using the Alexa Fluor 647 labelling kit (Life Technologies, UK) according to manufacturer’s recommendation prior to use. The CD107a degranulation of mononuclear cells were assessed following peptide stimulation or activation stimulation. The cells were incubated with relevant and irrelevant peptides or stimulated with Phorbol 12-myristate 13-acetate (PMA;50 ng/ml) and Ionomycin (Ion;1 μg/ml) in the presence of anti-CD107a antibody and incubated for 4 hours (41°C, 5% CO_2_). Following a wash in PBS, the cells were counter stained with anti-CD4 or anti-CD8_β_-PE (Cambridge Biotech, Cambridge, UK) for 15 min at 4°C and dead cells were excluded using 7-AAD-PE staining. The cells were acquired on a MACSQuant 10 flow cytometer (Miltenyi Biotec)and data were analysed using the Flow Jo software version 10 (Tree Star Inc).

### Real time-PCR of splenocytes stimulated with peptides

#### RNA extraction and cDNA

Total RNA was extracted from the splenocytes stimulated (41°C, 5% CO_2_) *ex vivo* with the peptides or PMA (50 ng/ml) plus Ion (1 μg/ml) for 18 hrs using TRIzol (Thermo Fisher Scientific, Paisley, UK) according to the manufacturer’s protocol. Subsequently, 1 µg of DNase treated, and purified RNA was reverse transcribed using a Superscript^®^ III First Strand Synthesis kit (Life technologies, Paisley, UK) and oligo-dT primers according to the manufacturer’s recommended protocol. The resulting cDNA was diluted at a ratio of 1:9 (cDNA:H_2_O) in Diethyl pyrocarbonate (DEPC)-treated water for use in Real Time-PCR assay.

#### SYBR green real time-PCR

Quantitative real-time PCR using SYBR Green was performed on the LightCycler^®^ 480 II (Roche Diagnostics GmbH, Mannheim, GER). Each reaction involved a pre-incubation at 95°C for 5 min, followed by 40 cycles of 95°C for 20 sec, 55°C–64°C (T_A_ as per primer); for 15 s, and elongation at 72°C for 10 s. Subsequent melt curve analysis was performed by heating to 95°C for 10 sec, cooling to 65°C for 1 min, and heating to 97°C. mRNA transcript levels of all genes were calculated as relative to the housekeeping gene β-actin using the LightCycler^®^ 480 Software (Roche Diagnostics GmbH, Mannheim, GER). To exclude the possibility of genomic DNA contamination, a no reverse transcriptase control (no RT) control was included in each template. The primer sequences used in this study are listed in **Table 1** (28, 29). Data represent mean of 6 biological replicates.

**Table 1:** List of primers used for Real-Time PCR.

Gene name	Accession no	Primers		Tm (^o^C)	Product size
Interleukin 2 (IL-2)	NM_204596.1	Fwd	ACAGTGGCTATAGGAGACGA	60**^o^C**	166
		Rev	TGTCTTGCTGGCTGTTGTGT		
Interleukin 4 (IL-4)	NM_001007079.1	Fwd	TGTGCCCACGCTGTGCTTACA	60**^o^C**	193
		Rev	CTTGTGGCAGTGCTGGCTCTCC		
Interleukin 10 (IL-10)	XM_025143715.1	Fwd	GGGAGCTGAGGGTGAAGTTTGAGGA	60**^o^C**	200
		Rev	CTGCTGATGACTGGTGCTGGTCTG		
Perforin	XM_004945690.3	Fwd	ATGGCGCAGGTGACAGTGA	60**^o^C**	271
		Rev	TGGCCTGCACCGGTAATTC		
Granzyme A (GZMA)	NM_204457.1	Fwd	TGGGTGTTAACAGCTGCTCATTGC	60**^o^C**	454
		Rev	CACCTGAATCCCCTCGACATGAGT		
Cytoplasmic Beta Actin (ACBT)	NM_205518.1	Fwd	TGCTGTGTTCCCATCTATCG	60**^o^C**	150 bp
		Rev	TTGGTGACAATACCGTGTTCA		

### Amino acid sequence alignment

In order to identify conserved putative T cell epitopes from different serotypes, the MDV strains (GAHV-2: RB-1B and CVI988, GAHV-3: SB-1, MEHV-1: HVT) pp38 (GenBank: ABR13155.1 for RB-1B strain; YP_001033989 for MD5 strain; ABF72309.1 for CVI988/Rispens strain; NP_066892.1 for HPRS24 strain; AEI00271.1 for SB-1 strain; NP_073357.1 for MEHV-1 strain; AAS01704.1 for MD11 strain; AAA46112.1 for GA strain) were aligned within and with each other, respectively. Clustal W was employed for the protein sequence alignment in MEGA6.

### Statistical analysis

ELISPOT SFU data were adjusted to 10^6^ cells. Quantification was performed using Graph Pad Prism 6 for windows. All data were analysed by one-way ANOVA or Wilcoxon and Mann Whitney non-parametric to test significance and presented as mean + Standard Deviation (SD). Results were considered statistically significant at *P* < 0.05 (*).

An immunological response/responder/reactivity was defined as a 2-fold increase in the frequency of cytokine-producing cells above control peptide/pools.

## Results

### Identification of distinct *pp38* derived immunodominant T cell epitopes in the resistant and susceptible chicken lines

**Figure 1:**
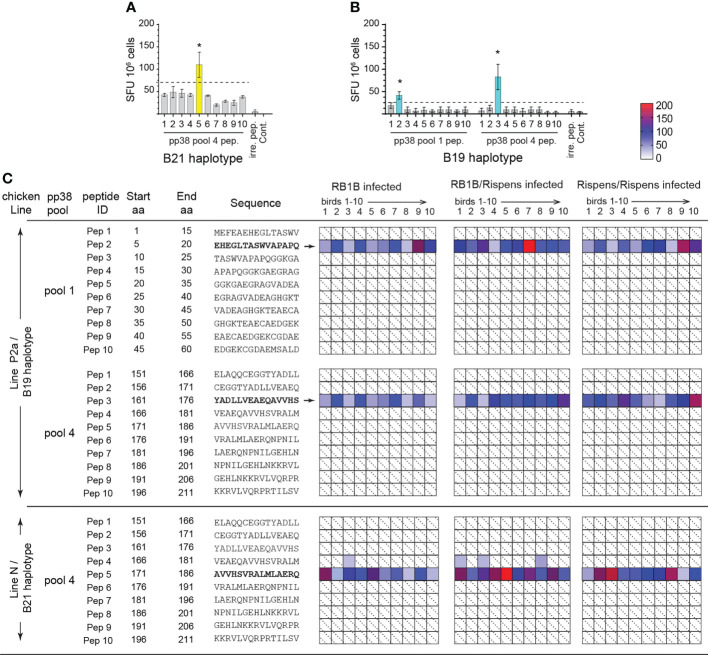
Identification of immunodominant pp38 derived T cell epitopes in B21 and B19 MHC haplotype chickens. T cells reactivity to individual 15 mer peptides spanning immunogenic regions of pp38 of MDV (1-60 sequence, peptide pool 1;151-211 sequence, peptide pool 4) determined in B21 MHC haplotype line N and B19 MHC haplotype line P2a chickens using an *ex vivo* ELISPOT assay for IFN-gamma. **(A, B)** Representative ELISPOT assay showing reactivity in **(A)** line N and **(B)** line P2a chickens to individual pp38 peptides within peptide pool 1 and 4 on week 2 post infection with MDV-RB1B. Bars represent the mean magnitude of chIFN-γ (SFU/10^6^ cells) *pp38-*specific T cell responses. **(C)** MDV *pp38-*specific T cell epitopes confirmed by single-peptide reactivity. Deconvoluted peptide pools screening each candidate reactive peptide present in a reactive row and reactive column; individual peptide response against individual chickens (1-10) in each group. Shaded in with dotted line boxes represent individual chickens that had no response to MDV *pp38-*peptides. Blue (line P2a) and yellow (line N) boxes indicate which is further defined by a heat map. *(*p* < 0.05) indicates a statistically significant difference. All assays were performed in triplicates.

*pp38* contains one immunogenic region (covering 151-211 sequence; peptide pool 4) capable of stimulating T cells in majority of the MD-resistant B21 MHC haplotype MDV-infected chickens. While two immunogenic regions (covering 1-60 and 151-211 sequences; peptide pool 1 and 4, respectively) are found to stimulate IFN-gamma from T cells isolated from majority of the MD-susceptible chickens following infection with virulent strain of MDV (RB1B) (26). To study MDV-specific T cell responses in line N (**Figure 1A**) or line P2a (**Figure 1B**) chickens, splenocytes were stimulated for 18h with individual peptides (15mer peptides with 10 overlapping amino acids), and the frequencies of IFN-gamma producing T cells were determined using an *ex vivo* ELISPOT assay for chicken IFN-gamma. IFN-gamma producing T cells recognizing regions of amino acid 1-60 (peptide pool 1) and amino acid 151-211 (peptide pool 4) within *pp38* sequence were induced in the vaccinated (Rispens) and/or MDV-infected (RB1B) B19 MHC haplotypes chickens (MD-susceptible line P2a) (**Figure 1C**), while this response was not detected in the non-infected naïve line P2a chickens. Similarly, T cells from the vaccinated (Rispens) and/or MDV-infected (RB1B) B21 MHC haplotype chickens (MD-resistant line N), but not naive birds, produced IFN-gamma in response to pp38 region of amino acid 1-60 (peptide pool 1) (**Figure 1C**). In total, T cell responses to the individual pp38 derived peptides were analysed in ten RB1B infected, ten vaccine- challenged (Rispens/RB1B), and ten vaccine-boost (Rispens/Rispens) birds from each line of chickens. In nearly all line P2a birds, the recognition frequency of pp38_5-20_ (EHEGLTASWVAPAPQ) and pp38_161-176_ (YADLLVEAEQAVVHS) based on peptide specific responses were 27/30 and 30/30 respectively. By contrast, all line N chickens recognized and responded to the pp38_171-186_ (AVVHSVRALMLAERQ) peptide. No T cell responses from vaccinated (Rispens) and/or MDV(RB1B)-infected line P2a and N birds were observed against the other pp38 pool 1 or pool 4 derived peptides (**Figures 1A–C**). None of the naïve control line P2a (n=10) or line N birds (n=10) produced IFN-gamma in response to the identified peptide epitopes.

**Figure 2:**
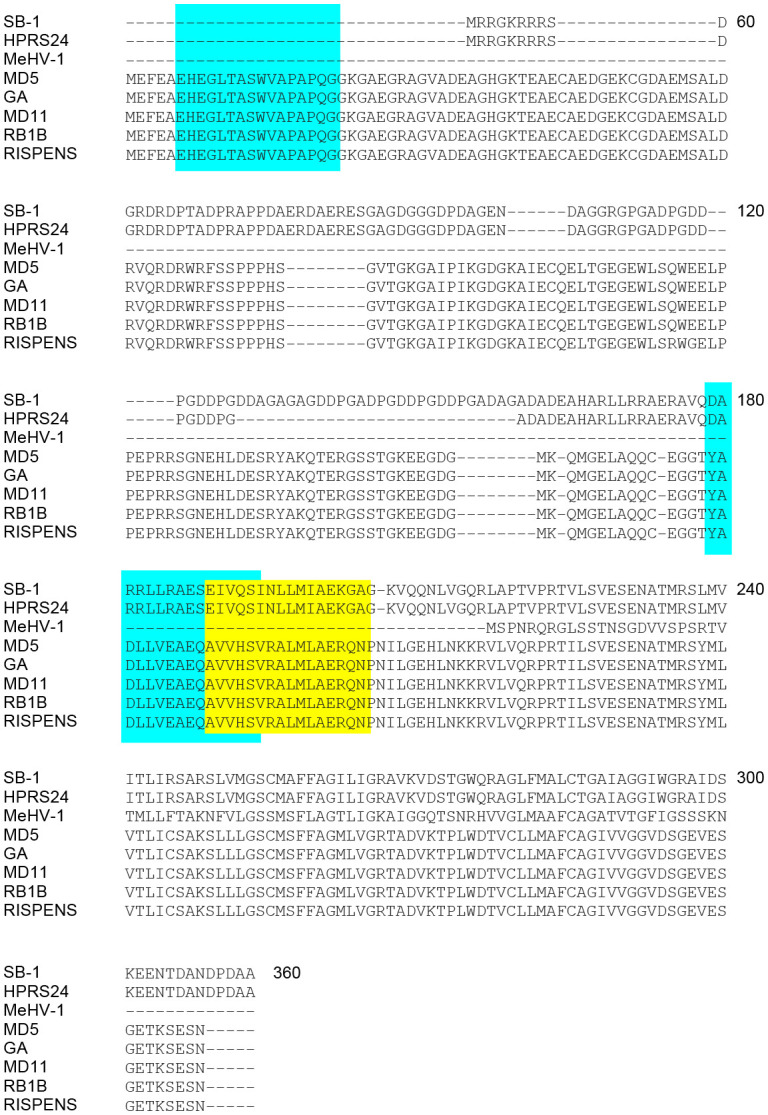
Alignment of MDV *pp38* proteins based on specific peptide sequences identified. Specific query of the identified immunodominant peptides was performed against all known avian viruses as well as MDV to assess whether these peptide sequences were conserved. **(A)** Alignment of *pp38* amino acid sequences from all known MDV serotypes (MDV-1; Rispens, RB1B, GA, MD11, and MD5, MDV-2; SB-1 and HPRS-24 and MDV-3; MeHV-1) was performed using MEGAX software. Blue boxes represent B19 haplotype specific peptide sequences (*pp38*_5-20_ and *pp38*_161-176_) and Yellow box represent the location of the B21 haplotype specific peptide sequence (*pp38*_171-186_). pp38 (GenBank: ABR13155.1 for RB-1B; YP_001033989 for MD5; ABF72309.1 for CVI988/Rispens; NP_066892.1 for HPRS24; AEI00271.1 for SB-1; NP_073357.1 for MEHV-1; AAS01704.1 for MD11; AAA46112.1 for GA).

Sequence alignment (NCBI SMARTBLAST tool; 2017) was performed based on GenBank deposited amino acid sequences for MDV *pp38* in the GaHV-2 (RB-1B, MD5, MD11, GA, CVI988-Rispens), GaHV-3 (SB-1 and HPRS24) and MeHV-1 (HVT/FC-126) strains (**Figure 2**). Blue and yellow highlighted regions correspond to the respective peptide sequences identified in line N (B21 haplotype) or line P2a (B19 haplotype) chickens. *pp38* peptide sequences (pp38_5-20_, pp38_161-176_ and pp38_171-186_) were confirmed to be highly conserved within MDV strains. Specifically, pp38_5-20_ (EHEGLTASWVAPAPQ) is conserved only within the GaHV-2 (MD5; YP_001033989.1, GA; AAF66817.1, MD11; AAS01704.1, RB1B; ABR13155.1 and CVI988-Rispens; AAB33524.1) strains. However, pp38_161-176_ (YADLLVEAEQAVVHS) and pp38_171-186_ (AVVHSVRALMLAERQ) were found to be highly conserved (15/15 aa) within the GaHV-2 strains shown in **Figure 2** and weakly conserved (6/15 aa) against GaHV-3 strains. Although the identified pp38_161-176_ and pp38_171-186_ share a short overlapping segment of 5 aa, our single peptide stimulation assay demonstrates that such difference are sufficient to discriminate between single peptides ability to elicit antigen specific T cell responses in line N or line P2a chickens. Sequence variations were noted in SB-1 (AEI00271.1), HPRS-24 (BAB16570.1) and HVT/FC-126 (NP_073357.1) with HVT/FC-126 containing a truncated pp38 protein. No *pp38* peptide sequence (pp38 _5-20_, pp38 _161-176_ and pp38 _171-186_) similarities were observed with HVT/FC-126. The identified antigenic regions are conserved within the GaHV-2 strains specifically to the vaccine (CVI988/Rispens) and the various pathogenic MDV strains (RB1B, MD5, MD11, GA) (**Figure 2**).

### CD4^+^TCR αβ/vβ_1_^+^ T cells from the MD-resistant and susceptible chicken lines recognize the immunodominant pp38-derived epitopes and produce IFN-gamma

**Figure 3:**
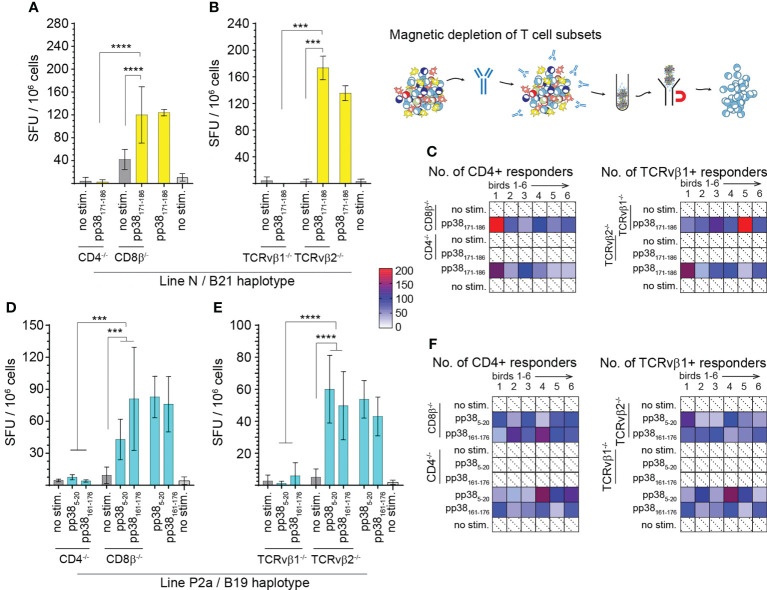
Virus-specific IFN-gamma producing CD4^+^TCR αβ/vβ_1_^+^ T cells. T cell subsets (CD4+, CD8β+, TCRvβ1+ or TCRvβ2+) were depleted from spleen mononuclear cells from line N **(A–C)** and line P2a **(D–F)** chickens by magnetic beads and the frequencies of pp38-derived peptide specific IFN-gamma producing T cells were determined using an *ex vivo* chIFN-γ ELISpot assay. Spot forming unit (SFU) in CD4^+^, CD8^+^ **(A, D)**, TCRvβ1^+^ or TCRvβ2^+^ T cells **(B, E)** are shown. Bars represent the mean magnitude of chIFN-γ (SFU/10^6^ cells) in response to specific *pp38*_5-20_, *pp38*_161-176_ and *pp38*_171-186_ peptide stimulation. Similar results were obtained in six line N **(C)** and six line P2a **(F)** chickens. Non-parametric Wilcoxon tests (Mann-Whitney) was used to assess normal distribution and test significance with the results shown as mean ± SD. *** (*p* < 0.001) and **** (*p* < 0.0001) indicates a statistically significant difference. All assays were performed in triplicates. All assays were performed in triplicates. Shaded in with dotted line boxes represent individual chickens that had no response to MDV *pp38-*peptides. Blue (Line P) and yellow (Line N) boxes indicate subject reactivity which is further defined by a heat map.

Subsequently, we confirmed the identity of the single peptides based on their respective ability to activate specific T cell subsets. CD4+, CD8+, TCRνβ_1_ or TCRνβ_2_+ cells were depleted from splenocytes from the MD-resistant B21 MHC haplotype line N (n=6) (**Figures 3A–C**) and MD-susceptible B19 MHC haplotype line P2a (n=6) chickens (**Figures 3D–F**). The frequencies of IFN-gamma producing T cells in response to the identified pp38-derived T cell epitopes (pp38_5-20_, pp38_161-176_ or pp38_171-186_) were determined using an *ex vivo* ELISPOT assay for chicken IFN-gamma. The results show that the splenocytes depleted of CD4^+^ or TCR2νβ_1_+ cells from line N (**Figures 3A–C**) or P2a (**Figures 3D–F**) chickens did not produce IFN-gamma in response to the corresponding identified pp38-derived T cell epitopes. In contrast, peptide stimulation elicited IFN-gamma production in the CD8β+ depleted, TCRνβ_2_+ depleted or non-depleted splenocytes of line N or line P2a chickens. Taken together, the results demonstrate that the region of amino acids 5-20 and 161-186 within pp38 protein contain epitopes that activated CD4+TCRνβ_1_+ T cells.

### *pp38*-specific CD4+ T cell from both the MD-resistant and susceptible chicken lines proliferate in a peptide specific manner

**Figure 4:**
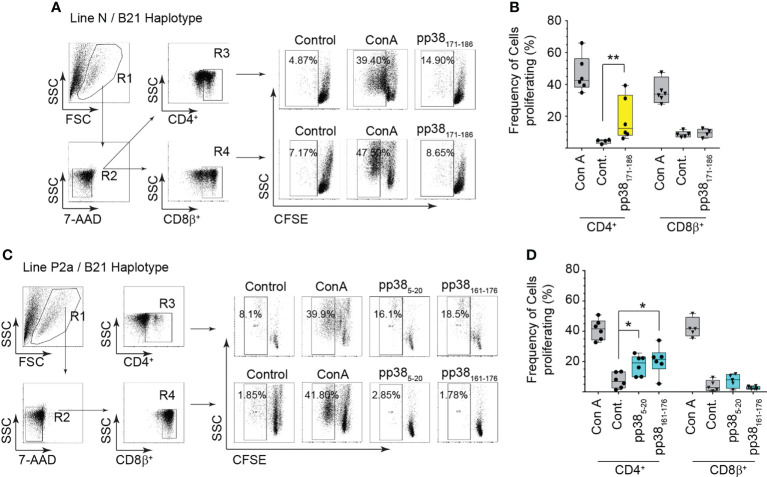
Virus-specific CD4+ T cell proliferation. Proliferation of CD 4 and CD8β T cells from the MDV-infected MD-resistant and susceptible chicken lines were analysed in response to the identified immunodominant peptides (*pp38*_5-20_, *pp38*_161-176_ and *pp38*_171-186_ peptide) using a CFSE-based T cell proliferation assay. Cells stimulated with Concanavalin A (Con A) (5ng/ml), diluent control (Cont.) or irrelevant peptide (influenza HA peptide: H5_246–260_) were used as controls. A representative dot plot and gating strategy to define **(A, C)** the percentage of proliferating cells within CD4+ or CD8+ T cells from **(B)** the MD-resistant B21 MHC haplotype line N **(D)** MD-susceptible B19 MHC haplotype line P2a are shown. Non-parametric Wilcoxon tests (Mann-Whitney) was used to assess normal distribution and test significance with the results shown as mean ± SD. *(*p* < 0.05) and **(*p* < 0.001) indicates a statistically significant difference. Data is presented as box and whiskers with bars representing the minimum and maximum distribution. All assays were performed in triplicates and results are representative of at least six chickens.

The ability of CD4+ and CD8_β_+ T cells from the MD-resistant B21 MHC haplotype line N (**Figures 4A, B**) and the MD-susceptible B19 MHC haplotype line P2a (**Figures 4C, D**) chickens to proliferate in response to the identified pp38-derived T cell epitopes were analyzed *in vitro* using a CFSE-based proliferation assay. Representative dot plots outlining the gating strategy for analysis of the proliferating T cells from the MDV-infected line N (MD-resistant) (**Figure 4A**) and line P2a (MD-susceptible) (**Figure 4C**) chickens are shown. The results demonstrate that both CD4+ and CD8_β_+ T cells from line N and line P2a chickens proliferate in response to Con A stimulation. In contrast, only CD4+ T cells, but not CD8β+ T cells, recognized the identified pp38-derived peptide epitopes (pp38_5-20_, pp38_161-176_ and pp38_171-186_) proliferated *in vitro*. There was no significant difference in the percentages of the proliferative cells between line N and line P2a chickens. Similarly, there was no differences in the proliferative abilities of pp38_161-176_ and pp38_5-20_ specific T cells from the MD-susceptible chickens (**Figures 4C, D**).

### *pp38*-specific expression of IL-2, IL-4, and Granzyme B genes in the MD-resistant chickens

**Figure 5:**
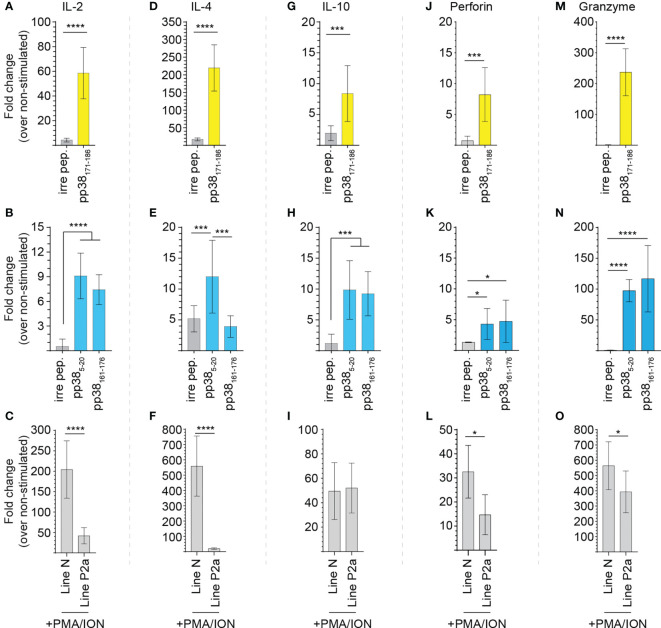
Virus-specific T cell cytokine expression. pp38-peptide specific and non-specific T cell cytokine expressions were determined in splenocytes of the MDV-infected MD-resistant and susceptible chicken lines using Real-Time PCR. Mononuclear cells were stimulated *ex vivo* with the *pp38*_5-20_, *pp38*_161-176_, *pp38*_171-186_, peptides, diluent control, or PMA plus ionomycin. Fold changes in the expressions of **(A–C)** IL-2, **(D–F)** IL-4, **(G–I)** IL-10, **(J–L)** perforin and **(M–O)** granzyme B genes were calculated over the non-stimulated. All assays were performed in triplicates and data are representative of 5 individual chickens from each chicken line. Yellow (MD-resistant line N) and blue (MD-susceptible line P2a) boxes indicate subject reactivity to listed peptides. *(*p* < 0.05), *** (*p* < 0.001) and **** (*p* < 0.0001) indicates a statistically significant difference.

*pp38*-specific and non-specific induction of chicken IL-2 (**Figures 5A–C**), IL-4 (**Figures 5D–F**), IL-10 (**Figures 5G–I**), perforin (**Figures 5J–L**) and granzyme B (**Figures 5M–O**) genes from splenocytes of the MDV-infected B21 MHC haplotype line N (MD-resistant; n=4) and B19 MHC haplotype line P2a (MD-susceptible; n=4) chickens were determined using qRT-PCR. Relative mRNA transcript levels induced by the identified *pp38* peptides (pp38_5-20_, *pp38*_161-176_ and pp38_171-186_) were compared with that induced by an irrelevant peptide (influenza HA peptide: H5_246–260_) over non-stimulated cells. Overnight pulsing with the identified epitopes elicited the induction of IL-2 (**Figures 5A, B**), IL-4 (**Figures 5D, E**), IL-10 (**Figures 5G, H**), perforin (**Figures 5J, K**) and granzyme B (**Figures 5M, N**) in both line N (labelled yellow) and P2a chickens (labelled blue) in a peptide-specific manner, respectively. Interestingly, *pp38*_171-186_ specific induction of IL-2, IL-4 and granzyme B in the MDV-infected line N chickens was significantly (p < 0.05) higher than that induced by *pp38*_5-20_ or *pp38*_161-176_ in the MDV-infected line P2a chickens. In contrast, no significant differences in induction of pp38 derived peptide-specific IL-10 or perforin mRNA transcripts were observed in the splenocytes of line N and line P2a chickens. To compare non-specific induction of IL-2, IL-4, IL-10, perforin and granzyme B genes in splenocytes from the MDV-infected line N and P2a chickens, cells were stimulated with PMA/Ion and the expression of these genes were analysed. The results demonstrate that splenocytes from MDV-infected B21 MHC haplotype line N chickens express higher levels of IL-2 (**Figure 5C**), IL-4 (**Figure 5F**), perforin (**Figure 5L**) and granzyme B (**Figure 5O**) compared to that induced in the B19 MHC haplotype line P2a. There was no difference in the expression of IL-10 between these two lines (**Figure 5I**). Interestingly, pp38_161-176_ specific T cells expressed higher levels of IL-4 compared to pp38_5-20_ specific T cells from the MD-susceptible chickens (**Figure 5E**).

### MDV-infection impairs virus-specific and non-specific CD4 and CD8 T cell degranulation (CD107a^+^) in both the MD-resistant and susceptible chicken lines

**Figure 6:**
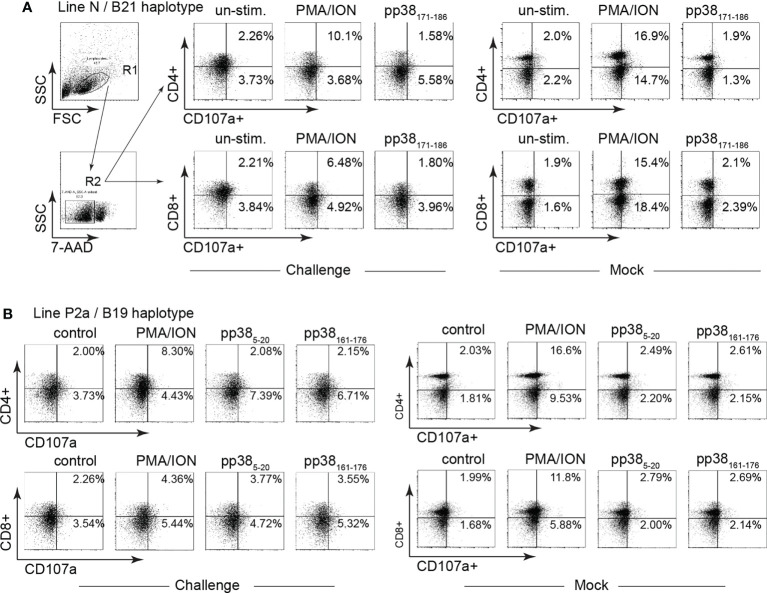
Gating strategy for identification of CD107a+ T cells. Degranulation (CD107a+) of CD4 and CD8β T cell subsets were determined in mononuclear cells isolated from the MD-resistant B21 MHC haplotype line N and MD-susceptible B19 MHC haplotype line P2a chickens in response to *pp38* peptides (*pp38*_5-20_, *pp38*_161-176_ and *pp38*_171-186_) or PMA plus Ionomycin stimulation. Representative dot plot and gating strategy to define CD4+CD107a+ and CD8+CD107a+ in **(A)** MD-resistant B21 MHC haplotype and **(B)** MD-susceptible B19 MHC haplotype line P2a chicken lines are shown.

**Figure 7:**
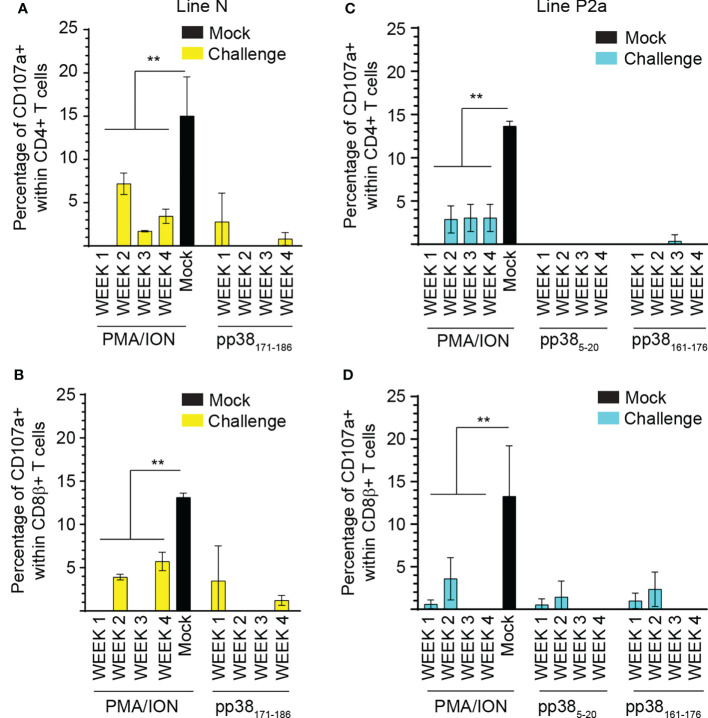
Impairment of T cell degranulation response following MDV infection. The percentages of **(A, C)** CD4+ and **(B, D)** CD8β+ αβ T cells expressing CD107a molecules in **(A, B)** MD-resistant B21 MHC haplotype line N **(C, D)** and MD-susceptible B19 MHC haplotype line P2a chickens are shown. Data are presented as percentage of CD107a+ cells stimulated with peptides or positive control subtracted from diluent stimulated control cells (background). Non-parametric Wilcoxon tests (Mann-Whitney) was used to assess normal distribution. Results are shown as mean ± SD. ** (*p* < 0.001) indicates a statistically significant difference. All assays were performed in triplicates and the data are representative of 5 chickens from each chicken lines.

Splenocytes from MDV-infected and mock-inoculated MD-resistant B21 MHC haplotype line N and MD-susceptible B19 MHC haplotype line P2a chickens were cultured with the identified *pp38*-derived peptide epitopes (*pp38*_171-186_ for MD-resistant line N; *pp38*_5-20_ and *pp38*_161-176_ for line P2a), cell culture media (unstimulated control) or PMA/ION for 6 hours. Degranulation of CD4+ and CD8β+ T cell was examined using CD107a expression by flow cytometry. Representative dot plots and gating strategy to define CD107a expression in CD4+ and CD8β + T cell in line N (**Figure 6A**) and line P2a (**Figure 6B**) are shown. The percentages of CD107a+ cells within CD4+ (**Figure 7A**) and CD8β + (**Figure 7B**) T cells of the MD-resistant line N are shown. The results demonstrate that pp38 _171-186_ does not induce degranulation in CD4+ or CD8β+ T cells from the MDV-infected or mock-inoculated line N chickens (**Figures 7B, C**). Similarly, stimulation of CD4+ (**Figure 7C**) or CD8β+ T cells (**Figure 7D**) from the MDV-infected or mock-inoculated MD-susceptible line P2a chickens with *pp*38 _5-20_ or *pp38*_161-176_ did not induce T cell degranulation. Interestingly, MDV infection reduced non-specific CD4+ and CD8β+ T cell degranulation in both the MD-resistant and susceptible chickens lines compared to that observed in the mock-inoculated control chickens (*p* < 0.01) (**Figures 7A–D**), indicating that T cell degranulation responses are impaired in MDV-infected chickens.

## Discussion

Two virus-specific pp38-derived immunodominant T cell epitopes in the MD-susceptible B19 MHC haplotype line P2a birds, and one immunodominant T cell epitopes in the MD-resistant B21 MHC haplotype line N birds are identified. Chicken CD4 or CD8 T cells either express TCRvβ1 or TCRvβ2 (30, 31), however, the contribution of these T cells in antigen-specific T cell response is yet unknown. Here, we demonstrated that the virus-specific CD4+TCRvβ1+ T cells from the MDV-infected chickens recognize the identified BL-B19 or BL-B21 restricted peptide epitopes and produce IFN-gamma. In this study, we aimed to identify an association between resistance to the disease and functional abilities of virus-specific effector T cells by analysing their virus-specific proliferative abilities, cytokine gene expression levels and degranulation responses. Analysis of the proliferative abilities of virus-specific T cells demonstrated that virus-specific CD4 T cells from both chicken lines can proliferate upon recognition of the identified pp38-derived peptide epitopes, even if virus-specific IL-2 gene expression was significantly higher in the MD-resistant chicken line. Interestingly, pp38-peptide specific activation also led to higher induction of IL-4 in the MD-resistant chicken line, suggesting an association between resistance to MD and virus-specific IL-2 and IL-4, but not IL-10, expressions. Thus, it seems that virus-specific effector T cells in the MD-resistant chickens express both Th1 and Th2 type responses, while virus-specific effector T cells in the MD-susceptible chickens is mainly skewed towards Th1 response. Non-specific stimulation of T cells also gave a similar cytokine pattern, indicating that there is a differential T cell cytokine expression in these birds. It is still unclear whether higher expression of IL-2 and IL-4 by pp38-specific or non-specific T cells from the MD-resistant chickens play any role in resistance to MD. IL-4 may have both tumour promoting and antitumour effects depending on the molecular and cellular environments, its sources, expressing time and dose (32). Further research is required to determine whether higher expressions of chicken IL-2 and IL-4 genes lead to higher synthesis of IL-2 and IL-4 proteins. The relationship between the levels of IL-2 and IL-4 transcripts and the amount of proteins translated in this environment requires further study. At the time of these experiments, validated assays for detection of chicken IL-2 and IL-4 proteins were not available to us. However, chIL-2 and chIL-4 ELISA assays are now commercially available.

In this study, expression of perforin and granzyme B genes were also upregulated by pp38 peptide-specific stimulation. Higher peptide-specific granzyme B expression was observed in the MD-resistant chickens compared to that in the MD-susceptible chickens. Similarly, non-specific stimulation induced higher levels of lymphocyte lysis-related genes, including granzyme B and perforin in the MDV-infected MD-resistant birds than that in the susceptible chicken line. This is in accordance with higher expression of IL-2 in the MD-resistant chickens, as it has been shown that IL-2 increases perforin and granzyme B expression (33). Conversely, IL-4 expression which is increases in the MD-resistant chickens, diminishes perforin/granzyme B expressions (34). It is possible, but not proven, that a balance between IL-2 and IL-4 expressions may determine the levels of perforin/granzyme B expression in the MD-resistant and susceptible chicken lines. Taken together, our results indicate that there is an association between expressions of perforin and granzyme B and resistance to the MD.

Finally, a CD107a mobilization assay was performed in virus specific and non-specific manner in both CD4 and CD8 T cells. Although cytotoxicity and perforin/granzyme B expression were once thought to be restricted to CD8 T cells, cytotoxicity of CD4 T cells, in a MHC class II restricted manner, have since been identified to play crucial roles in antiviral and antitumor immunity (35). Our results demonstrated that CD4 and CD8 T cell degranulation responses are impaired in both the MD-resistant and susceptible birds infected with MDV. Dysfunction of T cell cytotoxicity had been previously reported in chickens infected with chicken anaemia virus (36), while this is the first report showing that infection with a virulent strain of MDV impairs T cell cytotoxicity in naïve chickens. Vaccination of chickens against MDV may induce cytotoxic T cell response to lytic antigen pp38  (37), however, the role of vaccine-induced pp38-specific cytotoxic T cell response in the control of MD is currently unknown. It has been suggested that memory, but not effector, CD8 T cells can provide protection against infectious bronchitis virus (IBV) infection in the IBV-susceptible B19 haplotype chicken line (38). This is in accordance with our previous report demonstrating that there is an association between induction of MDV-specific IFN-gamma producing memory T cell response, but not effector T cell responses, and resistance to MD (26). Although, a weak virus-specific cytotoxic T cell response against several MDV antigens had been reported in chickens vaccinated against MDV (37, 39, 40), it is still unclear whether infection with a virulent MDV can also impair the vaccine-induced T cell cytotoxic response. This notion is supported by the results demonstrating that degranulation response of γδT cells in vaccinated/challenge group is significantly higher than that in the MDV-infected chickens (41). Further research is required to identify mechanisms involved in impairment of T cell degranulation/cytotoxicity response and determine whether this impairment can explain failure of vaccine-induced sterile immunity to control MDV replication and shedding from the infected birds. We have recently shown that the virulent MDV strain, but not vaccine strain of MDV, suppresses T cell proliferation *via* activation of the COX2/PGE2 pathway (10). It has been shown that PGE2 suppresses NK (42, 43) and γδ T cell cytotoxicity triggered by NKR and TCR through a cAMP-mediated PKA type I-dependent signalling (44). It is still unclear whether the activation of COX-2/PGE2 pathway by the virulent MDV contributes to impairment of T cell degranulation response in the MDV-infected chickens. As the vaccine strain of MDV (CVI988/Rispens) does not activate COX2/PGE2 pathway (10), further studies are required to determine whether (a) T cell degranulation responses in chickens infected with vaccine strain of MDV (CVI988/Rispens) are fully functional, and (b) infection of the vaccinated chickens with the virulent strain can impair T cell degranulation response. These experiments are planned with the aim to develop novel MDV vaccine which can induce sterile immunity.

In conclusion, the results demonstrate that the identified immunodominant MDV-derived peptide epitopes are recognized by IFN-gamma producing CD4+ TCRvβ1+ T cells in both the MD-resistant and susceptible chickens. However, functionally distinct T cells are induced in the MD-resistant chickens with higher expression levels of IL-4 and lymphocyte lysis-related genes compared to that in the MD-susceptible chickens. Importantly, the results demonstrate that MDV infection impairs T cell degranulation response in both the MD-resistant and susceptible chicken lines. Taken together, these data are important for our understanding of immune response against MDV and may pave away for development of more efficacious vaccines against MDV.

## Data availability statement

The original contributions presented in the study are included in the article/supplementary material. Further inquiries can be directed to the corresponding author.

## Ethics statement

Animal experiments were reviewed and approved by the ethical review committee at The Pirbright Institute (TPI) and the experiments were performed based on the guidelines and care approved by the UK government Home Office under project licence PPL 30/3169. The personnel engaged in the procedures had acquired personal license from the UK Home Office.

## Author contributions

Conceptualization: SB. Data curation: NB and SB. Formal analysis: NB and SB; Funding acquisition: SB. Investigation: NB and SB. Methodology: NB and SB. Supervision: SB Visualization: NB, SB. Writing—Original draft: NB and SB. Writing—Review and editing: SB. All authors contributed to the article and approved the submitted version.

## Funding

This work was supported by U.K. Research and Innovation Biotechnology and Biological Sciences Research Council Grants BBS/E/I/00001825, BBS/E/I/00007030, BBS/E/I/00007031, BB/S01506X/1, BBS/E/I/00002529, BBS/E/I/00007039, BBS/E/I/00007032, BB/N002598/1 and BB/V019031/1. The authors would also like to acknowledge the Pirbright Flow Cytometry facility and support through the Core capability grant (BBS/E/I/00007039).

## Acknowledgments

We acknowledge the excellent support from the Animal facilities at the Pirbright Institute.

## Conflict of interest

The authors declare that the research was conducted in the absence of any commercial or financial relationships that could be construed as a potential conflict of interest.

## Publisher’s note

All claims expressed in this article are solely those of the authors and do not necessarily represent those of their affiliated organizations, or those of the publisher, the editors and the reviewers. Any product that may be evaluated in this article, or claim that may be made by its manufacturer, is not guaranteed or endorsed by the publisher.
