# Corrigendum: Marek’s disease virus-specific T cells proliferate, express antiviral cytokines but have impaired degranulation response

**DOI:** 10.3389/fimmu.2022.1055936

**Published:** 2022-10-14

**Authors:** Nitish Boodhoo, Shahriar Behboudi

**Affiliations:** Avian Immunology, The Pirbright Institute, Woking, United Kingdom

**Keywords:** T cells, genetic resistance, genetic susceptibility, Marek’s disease virus, immunodominant epitopes

In the published article, there was an error in the **Funding** statement. Several fundings were not included. The correct **Funding** statement appears below.

“This work was supported by U.K. Research and Innovation Biotechnology and Biological Sciences Research Council Grants BBS/E/I/00001825, BBS/E/I/00007030, BBS/E/I/00007031, BB/S01506X/1, BBS/E/I/00002529, BBS/E/I/00007039, BBS/E/I/00007032, BB/N002598/1 and BB/V019031/1. The authors would also like to acknowledge the Pirbright Flow Cytometry facility and support through the Core capability grant (BBS/E/I/00007039).”

The authors apologize for this error and state that this does not change the scientific conclusions of the article in any way. The original article has been updated.

## Publisher’s note

All claims expressed in this article are solely those of the authors and do not necessarily represent those of their affiliated organizations, or those of the publisher, the editors and the reviewers. Any product that may be evaluated in this article, or claim that may be made by its manufacturer, is not guaranteed or endorsed by the publisher.

